# Systematic Bias Introduced by Genomic DNA Template Dilution in 16S rRNA Gene-Targeted Microbiota Profiling in Human Stool Homogenates

**DOI:** 10.1128/mSphere.00560-17

**Published:** 2018-03-14

**Authors:** Francesco Multinu, Sean C. Harrington, Jun Chen, Patricio R. Jeraldo, Stephen Johnson, Nicholas Chia, Marina R. Walther-Antonio

**Affiliations:** a Department of Obstetrics and Gynecology, Mayo Clinic, Rochester, Minnesota, USA; b Division of Biomedical Statistics and Informatics, Mayo Clinic, Rochester, Minnesota, USA; c Center for Individualized Medicine, Mayo Clinic, Rochester, Minnesota, USA; d Department of Physiology and Biomedical Engineering, Mayo Clinic, Rochester, Minnesota, USA; e Division of Surgery Research, Mayo Clinic, Rochester, Minnesota, USA; University of California, Merced

**Keywords:** 16S rRNA, gDNA, microbiome

## Abstract

Variability in representation of microbial communities can be caused by differences in microbial composition or artifacts introduced at sample collection or processing. Alterations in community representation introduced by variations in starting DNA concentrations have not been systematically investigated in stool samples. The goal of this study was to evaluate the effect of the genomic DNA (gDNA) concentration in the resulting 16S rRNA gene library composition and compare its effect to other sample processing variables in homogenized human fecal material. Compared to a gDNA input of 1 ng/μl, inputs of ≤1.6 × 10^−3^ ng/μl resulted in a marked decrease in the concentration of the 16S rRNA gene amplicon (*P* < 0.001). Low gDNA concentrations (≤1.6 × 10^−3^ ng/μl) were also associated with a decrease (*P* < 0.001) in the number of operational taxonomic units and significant divergence in β-diversity profiles (unweighted UniFrac distance, *P* < 0.001), as characterized by an overestimation of *Proteobacteria* and underestimation of *Firmicutes*. Even a gDNA concentration of 4 × 10^−2^ ng/μl showed a significant impact on the β-diversity profile (unweighted UniFrac distance, *P* = 0.03). Overall, the gDNA concentration explained 22.4% to 38.1% of the microbiota variation based on various β-diversity measures (*P* < 0.001). By comparison, the DNA extraction methods and PCR volumes tested did not significantly affect the microbial composition profile, and the PCR cycling method explained less than 3.7% of the microbiota variation (weighted UniFrac distance, *P* = 0.03). The 16S rRNA gene yield and the microbial community representation of human homogenized stool samples are significantly altered by gDNA template concentrations of ≤1.6 × 10^−3^ ng/μl. In addition, data from studies with a gDNA input of ≤4 × 10^−2^ ng/μl should be interpreted with caution.

**IMPORTANCE** The genomic DNA input for stool samples utilized for microbiome composition has not been determined. In this study, we determined the reliable threshold level under which conclusions drawn from the data may be compromised. We also determined the type of microbial bias introduced by less-than-ideal genomic input.

## Abstract (precis)

The genomic DNA input for stool samples utilized for microbiome composition has not been determined. In this study, we determined the reliable threshold level under which conclusions drawn from the data may be compromised. We also determined the type of microbial bias introduced by less-than-ideal genomic input.

## INTRODUCTION

Characterization of the microbial community present in biological samples (e.g., stool, urine, tissue, and blood) is becoming of paramount importance in the era of individualized medicine, with several studies showing that the complex community of microorganisms, which vary considerably between body sites and among individuals, has a key role in health and in disease (1, 2). The study of microbially diverse populations is routinely performed with 16S rRNA gene library construction and sequencing (3, 4), a protocol that requires an adequate microbial DNA concentration for accurate representation of the microbial community.

The effects of multiple variables on gut microbiome representation have been studied, and biases can be introduced through DNA extraction (4–6), PCR (7–10), and sequencing (11). For instance, PCR parameters such as the choice of primers and polymerase and the number of reaction cycles affect the richness (total number of species) and evenness (relative contribution of a given species to the total number of species) of the resulting sampling of the bacterial communities (4, 7–10). The impact of the template genomic DNA (gDNA) concentration in the community representation has been studied in sediment samples, where Wu et al. (8) reported that a dilution of from 20-fold to 200-fold reduced the determined taxon richness without having a significant impact on taxon evenness. In contrast, Biesbroek et al. (12) showed that diluting saliva DNA to below <1 pg/μl affected relative abundances and species representations, with an increased relative abundance of *Proteobacteria* and a significant decrease in the abundance of *Bacteroidetes* compared with identical samples with DNA concentrations above this threshold.

Because of the high concentration of native microbes in stool samples, the gDNA concentration typically is not a parameter of concern for evaluating gut microbiomes. However, this scenario can be affected by disease conditions such as diarrhea, recent antibiotic use, or chemotherapy drug use. Regardless, the initial gDNA concentrations, which often are not reported in publications, can potentially introduce relevant bias in study findings. In addition, dilution of stool gDNA templates has become a commonplace method to achieve optimal PCR efficiency and to dilute reaction inhibitors and host tissue DNA. However, the effect of the starting gDNA concentration in stool samples has not been studied. Similarly to the studies that established gDNA thresholds for accurate community representation in sediment and saliva samples, we sought to determine the level of input gDNA concentrations at which the microbial community becomes misrepresented in stool samples. We also studied the impact of different extraction methods and PCR conditions on the resulting microbial community profile.

## RESULTS

### Effects of technical variations on the overall microbiota profile.

**FIG 1:**
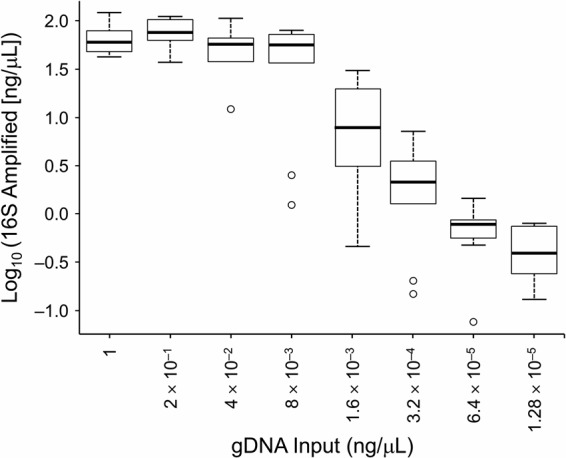
Values of correlations between genomic DNA (gDNA) reaction concentration and 16S rRNA gene yield (Spearman *r* = 0.83).

The total number of sequence reads was 6,888,783, with a sample range of 11,328 to 216,333. The median number of sequence reads was 70,601, and the median absolute deviation was 57,817. Among the tested variables, gDNA concentration and 16S rRNA gene yield had the strongest effects on the microbial community representation, explaining 22.4% to 38.1% and 20.7% to 42.8% of the overall microbiota variation, respectively, as summarized in Table 1. In contrast, the tested extraction methods (manual PowerSoil [Mo Bio Laboratories, Inc.] and automated Chemagic) and PCR volumes (25 and 50 μl) did not significantly affect microbial composition. PCR annealing temperatures (70°C annealing temperature and 69°C touchdown annealing temperature) significantly affected the microbiota representation but explained less than 3.7% of the community variation. Analysis of correlations between the 2 major predictors of the microbial profile (gDNA and 16S rRNA gene concentration) showed a high correlation (Spearman *r* = 0.83) (Fig. 1), suggesting that the effect of dilution on the microbial profile is likely mediated by the 16S rRNA gene yield.

**TABLE 1:** Marginal association between variables of interest and overall microbial composition^a^

Variable	Bray-Curtis distance		Unweighted UniFrac distance		Weighted UniFrac distance	
	*R*^2^ (%)	*P* value	*R*^2^ (%)	*P* value	*R*^2^ (%)	*P* value
gDNA concn (1 ng/μl to 1.28 × 10^−5^ ng/μl)	31.0	<0.001	38.1	<0.001	22.4	<0.001
16S rRNA gene yield	42.8	<0.001	39.5	<0.001	20.7	<0.001
Extraction method (Mo Bio vs Chemagic)	0.8	0.57	0.7	>0.79	0.6	0.78
PCR cycling method (standard vs touchdown)	1.1	0.37	1.5	0.23	3.7	0.03
PCR volume (25 vs 50 μl)	2.5	0.77	3.0	0.58	4.3	0.24

a Associations were assessed by using permutational multivariate analysis of variance, based on Bray-Curtis, unweighted UniFrac, and weighted UniFrac distances. Percentages of variance explained (*R*^2^) and *P* values are shown. Abbreviation: gDNA, genomic DNA.

### Overall diversity in microbiota composition associated with different levels of gDNA input.

**FIG 2:**
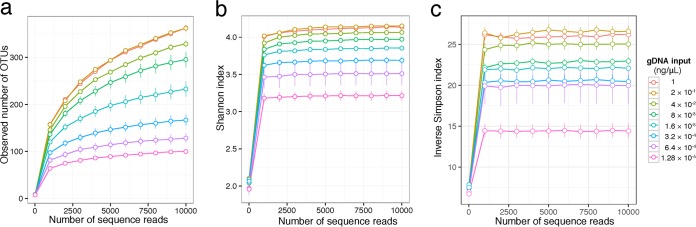
α-Diversity. Rarefaction curves comparing different concentrations of gDNA are shown. (a) Observed OTUs (species richness). (b) Shannon index. (c) Inverse Simpson index. gDNA, genomic DNA; OTU, operational taxonomic unit. Bars represent standard errors.

**FIG 3:**
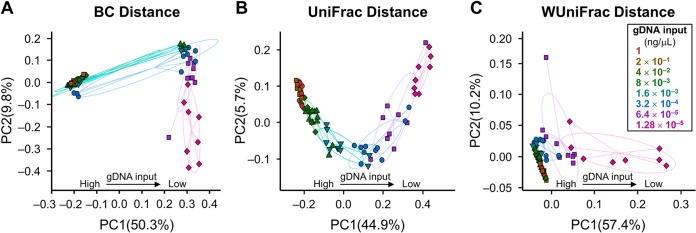
Principal-component (PC) plots showing β-diversity. (A) Bray-Curtis (BC) distance. (B) Unweighted UniFrac distance. (C) Weighted UniFrac (WUniFrac) distance. DNA reaction concentration levels are indicated. The axis labels show the percentages of variance explained by the PC analysis. gDNA, genomic DNA; OTU, operational taxonomic unit.

The levels of diversity in microbial composition observed with different levels of gDNA input were compared by assessing within-sample diversity (α-diversity) and between-sample diversity (β-diversity). The α-diversity, which was investigated by determining the number of observed operational taxonomic units (OTUs) (measure of sample richness) and the Shannon diversity index values (measure of sample richness and evenness), showed that low gDNA input was associated with lower overall diversity (Fig. 2). In particular, compared with a gDNA input of 1 ng/μl, a gDNA input of 8 × 10^−3^ ng/μl or lower was associated with a significant decrease in species richness (*P* < 0.001), whereas a gDNA input of 1.6 × 10^−3^ ng/μl or lower was associated with decreased overall diversity, as measured by the Shannon index (*P* < 0.001) (Table 2). Consistent with the α-diversity, the β-diversity analyses (Bray-Curtis, unweighted UniFrac, and weighted UniFrac distances) showed microbiota dissimilarity for different levels of gDNA input. Although principal-coordinate analyses based on all three β-diversity measures showed that a decrease in gDNA input was associated with a clear change in the representation of the microbiota (Fig. 3), the fact that the separation was more evident in the unweighted UniFrac analysis indicates that the major difference in microbiota occurs in the presence of and with an abundance of rare species (13) (Table 3). On the basis of the unweighted UniFrac distance data, a gDNA input of 4 × 10^−2^ ng/μl or lower resulted in a significant difference in the β-diversity profile compared with an input of 1 ng/μl. This difference was no longer significant if the gDNA input was at least 2 × 10^−1^ ng/μl.

**TABLE 2:** α-Diversity^a^

DNA reaction concn (ng/μl)	No. of observed OTUs, mean difference (*P* value)^b^	Shannon index, mean difference (*P* value)^c^	Inverse Simpson index, mean difference (*P* value)^d^
2 × 10^−1^	−0.664 (0.96)	0.0138 (0.88)	0.409 (0.78)
4 × 10^−2^	−32.1 (0.06)	−0.070 (0.43)	−1.15 (0.41)
8 × 10^−3^	−66.1 (<0.001)	−0.167 (0.07)	−3.22 (0.02)
1.6 × 10^−3^	−131 (<0.001)	−0.292 (0.002)	−4.11 (0.004)
3.2 × 10^−4^	−194 (<0.001)	−0.450 (<0.001)	−5.74 (<0.001)
6.4 × 10^−5^	−233 (<0.001)	−0.630 (<0.001)	−6.23 (<0.001)
1.28 × 10^−5^	−262 (<0.001)	−0.926 (<0.001)	−11.8 (<0.001)

a Comparison of each dilution with 1 ng/μl gDNA. Abbreviations: gDNA, genomic DNA; OTU, operational taxonomic units.

b The average number of observed OTUs was 362.

c The average Shannon index value was 4.14.

d The average inverse Simpson index value was 26.2.

**TABLE 3:** β-Diversity^a^

DNA reaction concn (ng/μl)	Bray-Curtis distance		Unweighted UniFrac distance		Weighted UniFrac distance	
	*R*^2^	*P* value	*R*^2^	*P* value	*R*^2^	*P* value
2 × 10^−1^	0.057	0.55	0.067	0.35	0.105	0.12
4 × 10^−2^	0.070	0.20	0.078	0.03	0.099	0.17
8 × 10^−3^	0.061	0.48	0.131	<0.001	0.275	<0.001
1.6 × 10^−3^	0.131	0.14	0.293	<0.001	0.402	<0.001
3.2 × 10^−4^	0.354	0.004	0.442	<0.001	0.251	<0.001
6.4 × 10^−5^	0.494	<0.001	0.473	<0.001	0.219	<0.001
1.28 × 10^−5^	0.442	<0.001	0.631	<0.001	0.486	<0.001

a Comparisons of each dilution with 1 ng/μl gDNA were based on permutational multivariate analysis of variance. Abbreviation: gDNA, genomic DNA.

### Specific microbial lineage bias associated with dilution.

**FIG 4:**
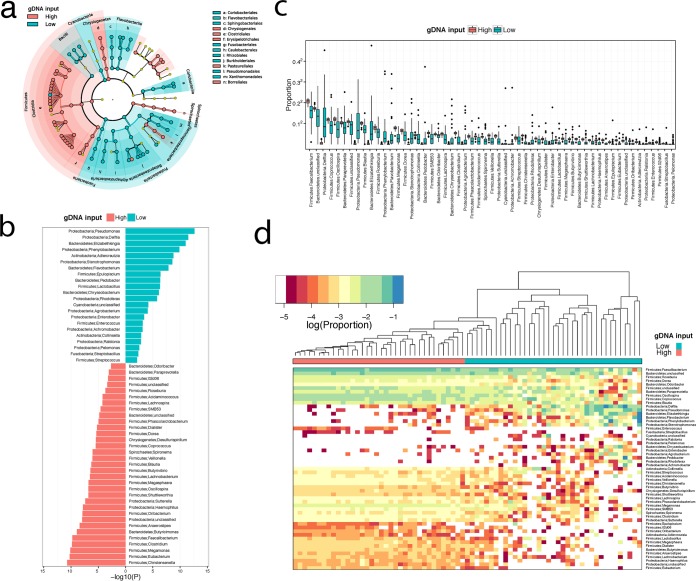
Different levels of gDNA input are associated with changes in microbial lineages. Samples with high and low gDNA reaction concentrations (ng/μl) were differentiated by using a cutoff value of 1.6 × 10^−3^ ng/μl. (a and b) Differentially abundant taxa (*q* value, < 0.01) are visualized on the phylogenetic tree (a), and their −log values (*P* values) are shown (b). Taxa enriched in the low-gDNA-concentration samples are indicated in blue, and taxa enriched in the high-gDNA-concentration samples are indicated in red. The genus *Pseudomonas* showed the most significant changes. (c) Differences in the proportions of the abundance of taxa between samples with high (red) and low (blue) gDNA reaction concentrations. (d) Heat map of the differentially abundant genera showed that the overall abundances of taxa differed between samples with high and low gDNA input. Samples with different gDNA concentration levels formed separate clusters (top; hierarchical clustering data are based on complete linkage and Euclidean distance). gDNA, genomic DNA.

Differential abundance analysis was performed to identify differentially abundant taxa at different levels of gDNA input. In particular, using 1.6 × 10^−3^ ng/μl of gDNA input as a cutoff, the analysis identified 55 differentially abundant genera (false-discovery-rate [*q*] value, <0.01) (Fig. 4a). The magnitude of difference for each of the taxa that were differentially abundant between samples with high gDNA input (>1.6 × 10^−3^ ng/μl) and low gDNA input (≤1.6 × 10^−3^ ng/μl) is indicated with the −log *P* value (Fig. 4b). Interestingly, the abundances of several genera of the phylum *Proteobacteria*, including *Pseudomonas*, *Delftia*, *Phenylobacterium*, and *Stenotrophomonas*, were highly overestimated in samples with low gDNA input. In contrast, numerous genera from the phylum *Firmicutes* (*Christensenella*, *Faecalibacterium*, *Clostridium*, *Megamonas*, *Eubacterium*) were underrepresented with low gDNA input (Fig. 4b). Moreover, the abundances of taxa of the low-gDNA-input group showed high variability (Fig. 4c). These differentially abundant genera could effectively separate the 2 groups, as revealed by hierarchical clustering based on the abundance profiles (Fig. 4d).

## DISCUSSION

In this study, we showed that template dilution and 16S rRNA gene yield had a significant effect on the representation of the microbial composition of stool samples. In particular, gDNA input at or below 8 × 10^−3^ ng/μl was associated with significantly lower within-sample diversity (α-diversity) and divergences in between-sample diversity (β-diversity). Moreover, template dilution was associated with a change in the microbial lineage, and a gDNA input level of 1.6 × 10^−3^ ng/μl or less was associated with overrepresentation of *Proteobacteria* and underrepresentation of *Firmicutes*. In particular, of the 22 taxa enriched by dilution, 10, namely, *Pseudomonas*, *Delftia*, *Stenotrophomonas*, *Flavobacterium*, *Pedobacter*, *Chryseobacterium*, *Enterobacter*, *Ralstonia*, *Pelomonas*, and *Streptococcus*, have been previously identified as common contaminants (14). This indicates that some of the bias introduced may relate to the increase in the contamination signal caused by the use of diluted template. However, the remaining results representing taxa overrepresented by dilution effects are likely unrelated to contaminating sources. A low gDNA concentration also increases the variability of the relative abundances and hence results in a larger measurement error. The tested DNA extraction methods (manual PowerSoil [Mo Bio, Inc.] and automated Chemagic), PCR annealing conditions (70°C and 69°C touchdown), and PCR volumes (25 and 50 μl) had no significant impact on the microbiome composition.

Previous studies evaluated the effect of template dilution on the microbial community representation of sediment samples (8) and saliva (12), but their results are not directly comparable to ours because of the high microbial abundance that characterizes stool samples. However, consistent with our findings, dilution of saliva was associated with an increase in the relative abundance of *Proteobacteria* (12).

To determine the impact of gDNA concentration on the accurate representation of the bacterial community and detection of rare species, which can be key components of a healthy or diseased community, we analyzed the α-diversity. Of the 2 α-diversity metrics used (number of observed OTU and Shannon index), the effect of reduced gDNA concentration on the number of observed OTUs was the more prominent, suggesting that the strongest effect is on species richness (detection of rare species) rather than on species evenness. Our results suggest that use of a gDNA concentration at or below 8 × 10^−3^ ng/μl would result in a significant underestimation of the number of species present and hence would influence the conclusions of the study.

To determine the potential bias introduced by gDNA concentration with respect to the variation between samples, we analyzed the β-diversity. Bias introduced at this level can artificially inflate sample divergence, leading to artificial groupings or diminishing the capacity to differentiate between truly divergent cohorts. We evaluated β-diversity with 3 metrics (Bray-Curtis, unweighted UniFrac, and weighted UniFrac distances) and showed that the effect of dilution was strongest on the unweighted UniFrac data, which capture the difference in rare and less-abundant species. This finding was consistent with what we observed with α-diversity, indicating that the loss in detection of rare species affects not only the within-sample diversity metrics but also the between-sample comparisons, potentially skewing results toward erroneous conclusions. The microbiota variability caused by different gDNA concentrations is therefore a critical criterion for consideration.

If DNA concentrations cannot be normalized among samples, particularly between cases and controls, statistical confounders may be created. More concerning, the gDNA concentration could confound the association of interest if it is also associated with the variable of interest. Unlike the findings of other studies (4, 5, 8), the effects of DNA extraction and PCR conditions on microbially diverse populations in our study were very limited, with DNA extraction not being a significant parameter and PCR conditions explaining less than 4% of the microbial variation. Both of these conclusions apply only to the variations that we have tested in both of these factors.

The present study had numerous strengths. To our knowledge, this was the first study to evaluate the effect of template dilution on the representation of microbial communities from stool samples. Because dilution has different effects (depending on microbial abundance) (12), these results will allow an improvement in the interpretation of the variation in the microbial community of stool samples. Moreover, because all fecal sample collection methods have been demonstrated to be reproducible, stable, and accurate (15), our results are generalizable to all studies evaluating fecal samples, independently of the collection methods. Another of the strengths of the study is that we used samples from the same homogenate, which eliminated the subject-to-subject variability that occurs in a population-based study and that would markedly affect variability (16). The study was thus powered to detect even very small effects of the technical variables evaluated. We note that the conclusions of this study are limited to the gDNA concentrations and processing conditions tested.

### Conclusion.

We performed a systematic and comprehensive assessment of the impact of various experimental parameters on the resulting 16S rRNA gene library and showed that microbial community representation can be affected by gDNA concentration and 16S rRNA gene yield. On the basis of these results, we recommend a minimum concentration above 4 × 10^−2^ ng/μl gDNA input, and, ideally, >2 × 10^−1^ ng/μl, to achieve an unbiased representation of the microbial community. The use of gDNA input levels of ≤1.6 × 10^−3^ ng/μl is not recommended because such levels would introduce taxonomic biases that would result in a definitive misrepresentation of the microbiome.

## MATERIALS AND METHODS

### Sample collection and homogenization.

Stool samples were prospectively collected from healthy volunteers (age, ≥18 years) at Mayo Clinic (Rochester, MN) from 4 September through 7 November 2014. Written informed consent for each subject was obtained following a protocol approved by the Mayo Clinic Institutional Review Board (protocol 13-005217). Participants were excluded from the study if they had received antibiotic or probiotic therapy within the 2 weeks before the collection date, had received chemotherapy, or had a history of pelvic radiation. Details of sample collection are reported elsewhere (15).

**FIG 5:**
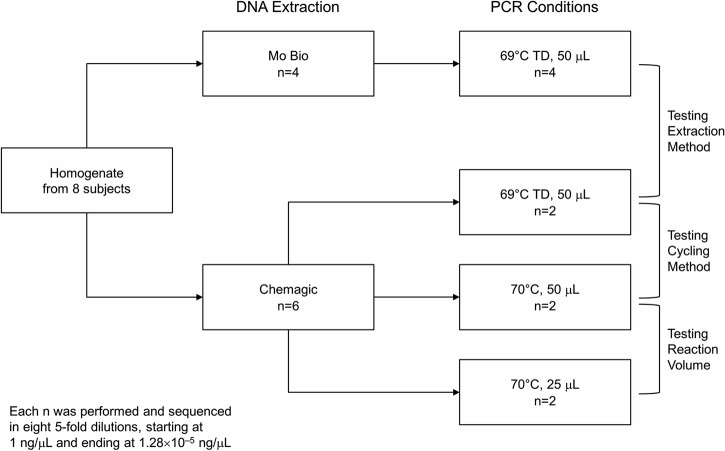
Study design. TD, touchdown.

For the present study, we used freshly collected stool from 8 patients; samples were combined with sterile phosphate-buffered saline and blended in a Stomacher 400 paddle blender (Seward Laboratory Systems Inc.). Each 150-μl aliquot of blended stool was placed in a 2-ml screw-cap tube (Starstedt; catalog no. 72.694.217) and stored at −80°C. The contents of one PowerBead tube (Mo Bio Laboratories, Inc.; catalog no. 12888-50-PBT) and 60 μl of solution C1 (Mo Bio Laboratories, Inc.; catalog no. 12888-50-1) were added to each aliquot. Aliquots were homogenized for 40 s with a FastPrep-24 instrument (MP Biomedicals LLC) at a speed setting of 6.0. Stool homogenates were clarified by centrifugation, pooled (2-ml total volume), and diluted to 3.4 ml with beading solution (Mo Bio Laboratories, Inc.; catalog no. 12888-50-BS). Four 800-μl aliquots were prepared from each homogenate pool and stored at −80°C before extraction testing. The study design is shown in Fig. 5.

### DNA extraction protocol and template dilution.

DNA extractions were performed in duplicate. Homogenate pools were thawed and added to a 96-well deep plate for automated DNA extraction with a Chemagic MSM I system or were processed manually with a PowerSoil DNA isolation kit (Mo Bio Laboratories, Inc.; catalog no. 12888-50) following the manufacturer’s protocol. DNA was eluted in 50-μl or 100-μl volumes for automated and manual processing, respectively. DNA concentrations were measured by the use of a Qubit fluorometer (Thermo Fisher Scientific), and total DNA yield was calculated based on elution volume.

From the homogenized fecal material and starting DNA concentration, the following eight 5-fold dilutions were prepared: 1 ng/μl, 2 × 10^−1^ ng/μl, 4 × 10^−2^ ng/μl, 8 × 10^−3^ ng/μl, 1.6 × 10^−3^ ng/μl, 3.2 × 10^−4^ ng/μl, 6.4 × 10^−5^ ng/μl, and 1.28 × 10^−5^ ng/μl.

### PCR parameters. (i) PCR cycling.

We tested 2 PCR cycling conditions. The first condition (*n* = 4) consisted of a 70°C annealing temperature (95°C for 5 min; 35 cycles of 98°C for 20 s, 70°C for 15 s, and 72°C for 15 s; and 72°C for 5 min). The second condition consisted of a 69°C touchdown annealing temperature (*n* = 8) (95°C for 5 min; 10 cycles of 98°C for 20 s, 70°C to 0.1°C [sequential decrease of 0.1°C per cycle] for 15 s, and 72°C for 15 s; 25 cycles of 98°C for 20 s, 69°C for 15 s, and 72°C for 15 s; and 72°C for 5 min). Both reactions included 35 amplification cycles.

### (ii) PCR volume.

We tested 2 PCR volumes: 25 μl and 50 μl.

Amplification with Kapa HiFi Hotstart Ready Mix (Kapa Biosystems) polymerase targeted V3 and V5 regions of the bacterial 16S rRNA gene subunit (8) using primers 357 F (5′-AATGATACGGCGACCACCGAGATCTACACTATGGTAATTGTCCTACGGGAGGCAGCAG-3′) and 926R (5′-CAAGCAGAAGACGGCATACGAGATNNNNNNNNNNNNAGTCAGTCAGCCCCGTCAATTCMTTTRAGT-3′), with barcodes from Caporaso et al. (17). A unique 12-nucleotide error-correcting Golay barcode was used to label each amplicon (represented by N's). The target amplicon size (approximately 700 bp) was verified by using a 2200 TapeStation instrument (Agilent Technologies). DNA was purified with Agencourt AMPure (Beckman Coulter, Inc.).

### Sequencing.

The 16S rRNA gene sequencing was performed using a next-generation Illumina MiSeq 300-bp read sequencing platform with custom sequencing primers read1 (R1; 5′-TATGGTAATTGTCCTACGGGAGGCAGCAG-3′), read2 (R2; 5′-AGTCAGTCAGCCCCGTCAATTCMTTTRAGT-3′), and index (5′-ACTYAAAKGAATTGACGGGGCTGACTGACT-3′). R1s and R2s were first demultiplexed by the use of Illumina BCL2Fastq software. Paired R1-R2 reads were analyzed using *hybrid-denovo* (18), which was specifically developed for nonoverlapping paired-end reads, with default parameter settings. Briefly, quality filtering was performed using Trimmomatic (19). Surviving read pairs were further trimmed, concatenated, and sorted by cluster size. Afterward, *de novo* operational taxonomic unit (OTU) picking (97% similarity) was conducted via the use of the UPARSE algorithm (20). UCHIME was used to perform additional reference-based chimera removal against the gold database (21). High-quality single-end R1s were further mapped to the R1 end of the OTU representatives using USEARCH (22). The remaining unmapped R1s were clustered into new OTUs via the use of the UPARSE algorithm and added to the list of OTUs generated by the paired-end reads. Thus, the OTU representatives consisted of a mixture of single-end and paired-end reads. We then aligned all the OTU representatives using the structure alignment algorithm Infernal trained on the Ribosomal Database Project (RDP) database (23). OTU representatives that were not aligned or that had negative alignment scores were removed since they hypothetically represented nonbacteria. A phylogenetic tree was built from the aligned OTU representatives using FastTree (24). FastTree has a low penalty level with respect to end gaps, which is favorable when processing a mixture of single-end and paired-end reads. Finally, R1 and R2 reads were stitched together with ambiguous nucleotides between them and then assigned taxonomy data by the RDP classifier (25) trained on the Greengenes database v13.5 (26). OTUs not classified as *Bacteria* and singleton OTUs were removed as they were presumed to be contaminants. Samples with fewer than 2,000 reads were removed.

### Statistical analysis.

We investigated the effects of technical variables on the overall microbiota structure as described previously (27). We first summarized the microbiota data by using α-diversity and β-diversity measures. Three α-diversity metrics were used: the observed OTU number, the Shannon index, and the inverse Simpson index. The observed OTU number reflects species richness, whereas the Shannon and inverse Simpson index values take into account both species richness and species evenness. Inverse Simpson index values place more weight on dominant taxa than Shannon index values. β-Diversity values, in contrast, indicate the levels of diversity shared by bacterial populations in terms of ecological distance. Different distance metrics provide distinctive views of community structure. Three β-diversity measures, namely, Bray-Curtis, unweighted UniFrac, and weighted UniFrac distances, were calculated by using the OTU table and a phylogenetic tree (“vegdist” and “GUniFrac” function in the R packages “vegan” and “GUniFrac”). Analyses that determine weighted UniFrac and unweighted UniFrac distance values are designed to detect phylogenetic signals, i.e., clusters of OTUs on the phylogenetic tree, while Bray-Curtis distance values are more efficient in catching scattered signals. Weighted UniFrac distance values place more weight on the significance of abundant lineages and are more efficient at detecting changes in abundant lineages, while unweighted UniFrac distance values use presence and absence information and are powerful for detecting community membership change or changes in abundance in rare lineages (if the abundance is below the detection limit, it will appear to represent absence) (13). Significant values achieved using any of the statistical metrics described above are reported in the manuscript. Sequencing data were rarefied to a common depth of 11,328 reads before α-diversity and β-diversity analyses were performed.

To assess the association between a technical variable and α-diversity, we fitted a linear regression model to the α-diversity metrics after rarefaction. gDNA concentration and 16S rRNA gene yield data were log-transformed before analysis. To assess the association with β-diversity measures, we used permutational multivariate analysis of variance (PERMANOVA; “Adonis” function in the R “vegan” package). Significance was assessed with 1,000 permutations. A distance-based *R*^2^ value was used to quantify the percentage of variability of the microbiota that was explained by various technical factors (28). Ordination plots based on Bray-Curtis, unweighted UniFrac, and weighted UniFrac distances were generated with principal-coordinate analysis as implemented in R (“cmdscale” function in the R “stats” package). To identify bacterial genera associated with gDNA concentration, we conducted differential abundance analysis at the genus level by using the Wilcoxon rank sum test. We filtered out genera with a prevalence of less than 10%. We normalized the count data into relative abundances (proportions) by dividing by the total read count. Taxa with a maximum proportion of less than 0.2% were excluded from testing to reduce the number of the tests. False-discovery-rate control (B-H procedure; “padjust” in standard R packages) was used to correct for multiple testing, and false-discovery-rate-adjusted *P* values (*q* values) of less than 0.01 were considered significant.

### Data availability.

The sequencing data sets are available in the National Center for Biotechnology Information repository (study ID SRP098583).

## References

[1] Schwabe RF, Jobin C 2013 The microbiome and cancer. Nat Rev Cancer 13:800–812. doi:10.1038/nrc3610. 24132111 PMC3986062

[2] White BA, Creedon DJ, Nelson KE, Wilson BA 2011 The vaginal microbiome in health and disease. Trends Endocrinol Metab 22:389–393. doi:10.1016/j.tem.2011.06.001. 21757370 PMC3183339

[3] Weinstock GM 2012 Genomic approaches to studying the human microbiota. Nature 489:250–256. doi:10.1038/nature11553. 22972298 PMC3665339

[4] Henderson G, Cox F, Kittelmann S, Miri VH, Zethof M, Noel SJ, Waghorn GC, Janssen PH 2013 Effect of DNA extraction methods and sampling techniques on the apparent structure of cow and sheep rumen microbial communities. PLoS One 8:e74787. doi:10.1371/journal.pone.0074787. 24040342 PMC3770609

[5] Gerasimidis K, Bertz M, Quince C, Brunner K, Bruce A, Combet E, Calus S, Loman N, Ijaz UZ 2016 The effect of DNA extraction methodology on gut microbiota research applications. BMC Res Notes 9:365. doi:10.1186/s13104-016-2171-7. 27456340 PMC4960752

[6] Inceoglu O, Hoogwout EF, Hill P, van Elsas JD 2010 Effect of DNA extraction method on the apparent microbial diversity of soil. Appl Environ Microbiol 76:3378–3382. doi:10.1128/AEM.02715-09. 20348306 PMC2869127

[7] Sergeant MJ, Constantinidou C, Cogan T, Penn CW, Pallen MJ 2012 High-throughput sequencing of 16S rRNA gene amplicons: effects of extraction procedure, primer length and annealing temperature. PLoS One 7:e38094. doi:10.1371/journal.pone.0038094. 22666455 PMC3362549

[8] Wu JY, Jiang XT, Jiang YX, Lu SY, Zou F, Zhou HW 2010 Effects of polymerase, template dilution and cycle number on PCR based 16 S rRNA diversity analysis using the deep sequencing method. BMC Microbiol 10:255. doi:10.1186/1471-2180-10-255. 20937143 PMC2964677

[9] Pinto AJ, Raskin L 2012 PCR biases distort bacterial and archaeal community structure in pyrosequencing datasets. PLoS One 7:e43093. doi:10.1371/journal.pone.0043093. 22905208 PMC3419673

[10] Engelbrektson A, Kunin V, Wrighton KC, Zvenigorodsky N, Chen F, Ochman H, Hugenholtz P 2010 Experimental factors affecting PCR-based estimates of microbial species richness and evenness. ISME J 4:642–647. doi:10.1038/ismej.2009.153. 20090784

[11] Clooney AG, Fouhy F, Sleator RD, O’ Driscoll A, Stanton C, Cotter PD, Claesson MJ 2016 Comparing apples and oranges?: next generation sequencing and its impact on microbiome analysis. PLoS One 11:e0148028. doi:10.1371/journal.pone.0148028. 26849217 PMC4746063

[12] Biesbroek G, Sanders EA, Roeselers G, Wang X, Caspers MP, Trzciński K, Bogaert D, Keijser BJ 2012 Deep sequencing analyses of low density microbial communities: working at the boundary of accurate microbiota detection. PLoS One 7:e32942. doi:10.1371/journal.pone.0032942. 22412957 PMC3295791

[13] Chen J, Bittinger K, Charlson ES, Hoffmann C, Lewis J, Wu GD, Collman RG, Bushman FD, Li H 2012 Associating microbiome composition with environmental covariates using generalized UniFrac distances. Bioinformatics 28:2106–2113. doi:10.1093/bioinformatics/bts342. 22711789 PMC3413390

[14] Salter SJ, Cox MJ, Turek EM, Calus ST, Cookson WO, Moffatt MF, Turner P, Parkhill J, Loman NJ, Walker AW 2014 Reagent and laboratory contamination can critically impact sequence-based microbiome analyses. BMC Biol 12:87. doi:10.1186/s12915-014-0087-z. 25387460 PMC4228153

[15] Vogtmann E, Chen J, Amir A, Shi J, Abnet CC, Nelson H, Knight R, Chia N, Sinha R 2017 Comparison of collection methods for fecal samples in microbiome studies. Am J Epidemiol 185:115–123. doi:10.1093/aje/kww177. 27986704 PMC5253972

[16] Falony G, Joossens M, Vieira-Silva S, Wang J, Darzi Y, Faust K, Kurilshikov A, Bonder MJ, Valles-Colomer M, Vandeputte D, Tito RY, Chaffron S, Rymenans L, Verspecht C, De Sutter L, Lima-Mendez G, D’hoe K, Jonckheere K, Homola D, Garcia R, Tigchelaar EF, Eeckhaudt L, Fu J, Henckaerts L, Zhernakova A, Wijmenga C, Raes J 2016 Population-level analysis of gut microbiome variation. Science 352:560–564. doi:10.1126/science.aad3503. 27126039

[17] Caporaso JG, Kuczynski J, Stombaugh J, Bittinger K, Bushman FD, Costello EK, Fierer N, Peña AG, Goodrich JK, Gordon JI, Huttley GA, Kelley ST, Knights D, Koenig JE, Ley RE, Lozupone CA, McDonald D, Muegge BD, Pirrung M, Reeder J, Sevinsky JR, Turnbaugh PJ, Walters WA, Widmann J, Yatsunenko T, Zaneveld J, Knight R 2010 QIIME allows analysis of high-throughput community sequencing data. Nat Methods 7:335–336. doi:10.1038/nmeth.f.303. 20383131 PMC3156573

[18] Chen X, Johnson S, Jeraldo P, Wang J, Chia N, Kocher J-PA, Chen J 15 12 2017 Hybrid-denovo: a *de novo* OTU-picking pipeline integrating single-end and paired-end 16S sequence tags. GigaScience https://academic.oup.com/gigascience/advance-article/doi/10.1093/gigascience/gix129/4750782. 10.1093/gigascience/gix129 PMC5841375 29267858

[19] Bolger AM, Lohse M, Usadel B 2014 Trimmomatic: a flexible trimmer for Illumina sequence data. Bioinformatics 30:2114–2120. doi:10.1093/bioinformatics/btu170. 24695404 PMC4103590

[20] Edgar RC 2013 Uparse: highly accurate OTU sequences from microbial amplicon reads. Nat Methods 10:996–998. doi:10.1038/nmeth.2604. 23955772

[21] Edgar RC, Haas BJ, Clemente JC, Quince C, Knight R 2011 UCHIME improves sensitivity and speed of chimera detection. Bioinformatics 27:2194–2200. doi:10.1093/bioinformatics/btr381. 21700674 PMC3150044

[22] Edgar RC 2010 Search and clustering orders of magnitude faster than BLAST. Bioinformatics 26:2460–2461. doi:10.1093/bioinformatics/btq461. 20709691

[23] Nawrocki EP, Kolbe DL, Eddy SR 2009 Infernal 1.0: inference of RNA alignments. Bioinformatics 25:1335–1337. doi:10.1093/bioinformatics/btp157. 19307242 PMC2732312

[24] Price MN, Dehal PS, Arkin AP 2010 FastTree 2–approximately maximum-likelihood trees for large alignments. PLoS One 5:e9490. doi:10.1371/journal.pone.0009490. 20224823 PMC2835736

[25] Wang Q, Garrity GM, Tiedje JM, Cole JR 2007 Naive Bayesian classifier for rapid assignment of rRNA sequences into the new bacterial taxonomy. Appl Environ Microbiol 73:5261–5267. doi:10.1128/AEM.00062-07. 17586664 PMC1950982

[26] DeSantis TZ, Hugenholtz P, Larsen N, Rojas M, Brodie EL, Keller K, Huber T, Dalevi D, Hu P, Andersen GL 2006 Greengenes, a chimera-checked 16S rRNA gene database and workbench compatible with ARB. Appl Environ Microbiol 72:5069–5072. doi:10.1128/AEM.03006-05. 16820507 PMC1489311

[27] Hieken TJ, Chen J, Hoskin TL, Walther-Antonio M, Johnson S, Ramaker S, Xiao J, Radisky DC, Knutson KL, Kalari KR, Yao JZ, Baddour LM, Chia N, Degnim AC 2016 The microbiome of aseptically collected human breast tissue in benign and malignant disease. Sci Rep 6:30751. doi:10.1038/srep30751. 27485780 PMC4971513

[28] Chen J, Ryu E, Hathcock M, Ballman K, Chia N, Olson JE, Nelson H 2016 Impact of demographics on human gut microbial diversity in a US Midwest population. PeerJ 4:e1514. doi:10.7717/peerj.1514. 26839739 PMC4734456

